# Recent Advances in Utilizing Transcription Factors to Improve Plant Abiotic Stress Tolerance by Transgenic Technology

**DOI:** 10.3389/fpls.2016.00067

**Published:** 2016-02-09

**Authors:** Hongyan Wang, Honglei Wang, Hongbo Shao, Xiaoli Tang

**Affiliations:** ^1^Institute of Technology, Yantai Academy of China Agriculture UniversityYantai, China; ^2^Jiangsu Key Laboratory for Bioresources of Saline Soils, Provincial Key Laboratory of Agrobiology, Institute of Biotechnology, Jiangsu Academy of Agricultural SciencesNanjing, China; ^3^Key Laboratory of Coastal Biology and Bioresources Utilization, Yantai Institute of Coastal Zone Research, Chinese Academy of SciencesYantai, China

**Keywords:** abiotic stress, transcription factors, transgenic plant, stress-responsive, stress tolerance

## Abstract

Agricultural production and quality are adversely affected by various abiotic stresses worldwide and this will be exacerbated by the deterioration of global climate. To feed a growing world population, it is very urgent to breed stress-tolerant crops with higher yields and improved qualities against multiple environmental stresses. Since conventional breeding approaches had marginal success due to the complexity of stress tolerance traits, the transgenic approach is now being popularly used to breed stress-tolerant crops. So identifying and characterizing the critical genes involved in plant stress responses is an essential prerequisite for engineering stress-tolerant crops. Far beyond the manipulation of single functional gene, engineering certain regulatory genes has emerged as an effective strategy now for controlling the expression of many stress-responsive genes. Transcription factors (TFs) are good candidates for genetic engineering to breed stress-tolerant crop because of their role as master regulators of many stress-responsive genes. Many TFs belonging to families AP2/EREBP, MYB, WRKY, NAC, bZIP have been found to be involved in various abiotic stresses and some TF genes have also been engineered to improve stress tolerance in model and crop plants. In this review, we take five large families of TFs as examples and review the recent progress of TFs involved in plant abiotic stress responses and their potential utilization to improve multiple stress tolerance of crops in the field conditions.

## Introduction

Agricultural production and quality are adversely affected by a broad range of abiotic stresses including drought, salinity, heat, and cold. Especially when these stresses occur in combination, it can have devastating effects on plant growth and productivity. It is estimated that more than 50% of worldwide yield loss for major crop are caused by abiotic stresses (Shao et al., 2009; Ahuja et al., 2010; Lobell et al., 2011). According to the current climate prediction models, the deterioration of global climate will inevitably cause an increased frequency of drought, heat wave, and salinization (Easterling et al., 2000; Ipcc, 2007, 2008). This means that agricultural productivity will face a greater challenge in fighting against environmental stresses. Meanwhile, the growing world population will reach close to ten billion by the year 2050 and then almost two times of current agricultural productivity is needed to feed the large population (Bengtsson et al., 2006; United Nations, 2015). Moreover, such a tremendous increase of crop productivity must be achieved with no increase in arable land and in the face of multiple environmental stresses. Where is the way to solve this problem? Many scholars and experts worldwide have reached a consensus that breeding stress-tolerant crops with higher yields and improved qualities against multiple environmental stresses is an effective strategy, as well as one of the greatest challenges faced by modern agriculture (Takeda and Matsuoka, 2008; Newton et al., 2011; Liu J.-H. et al., 2014). In the past few decades, a great deal of efforts has been devoted to breeding crop cultivars with various stress-tolerant traits. Two main approaches have been employed to this process. One is traditional breeding methods such as wide-cross hybridization and mutation breeding, which often brings about unpredictable results. Another is modern transgenic technology by introducing novel exogenous genes or altering the expression levels of endogenous genes to improve stress tolerance. Since conventional breeding approaches have marginal success due to the complexity of stress tolerance traits, the transgenic approach is now being popularly used to develop transgenic crops tolerant to abiotic stresses (Yamaguchi and Blumwald, 2005). Therefore, deciphering the molecular mechanisms by which plants perceive and transduce stress signals to cellular machinery to initiate adaptive responses is an essential prerequisite for identification of the key genes and pathways to engineer stress-tolerant crop plants (Ray et al., 2009; Heidarvand and Amiri, 2010; Sanchez et al., 2011).

Substantial progress has been made to unravel the molecular mechanisms of abiotic stress responses in plants by means of high throughput sequencing and functional genomics tools. To date, a number of critical genes involved in abiotic stress tolerance have been identified and validated, which are generally classified into two types: functional genes and regulatory genes (Shinozaki et al., 2003). The former encodes important enzymes and metabolic proteins (functional proteins), such as detoxification enzyme, water channel, ion transporter, heat shock protein (HSP), and late embryogenesis abundant (LEA) protein, which directly function to protect cells from stresses. The latter encodes various regulatory proteins including TFs, protein kinases and protein phosphatases, which regulate signal transduction and gene expression in the stress responses. Although there have been numerous studies on functional genes, most of these studies pay more attention to single gene or several genes encoding enzymes and protective proteins by imposing a given stress. Due to the complexity of stress responses regulated by multi-genes, little success has been achieved by a single functional gene approach to significantly enhance plant stress tolerance (Mittler and Blumwald, 2010; Varshney et al., 2011). Given the complexity and variability of field conditions where crops are often simultaneously subjected to multiple abiotic stresses or some in combination (Ahuja et al., 2010), more and more studies have paid close attention to regulatory genes and found that some regulatory genes including TFs play essential roles in multiple abotic stress responses by regulating a large spectrum of downstream stress-responsive genes. Thus, genetically modifying the expression of certain regulatory genes can greatly influence plant stress tolerance because it mimics or enhances stress signals to regulate many downstream stress-responsive genes at a time (Century et al., 2008; Yang et al., 2011). Among the regulatory genes, stress-responsive TFs have attracted particular attention due to their important roles in plant stress responses (Chen and Zhu, 2004; Xu et al., 2008a). In this paper, we mainly review the recent progress of TFs involved in plant abiotic stress responses and their potential utilization to improve multiple stress tolerance of crops in the field conditions.

## The generic signaling pathway involved in plant abiotic stress responses

**Figure 1:**
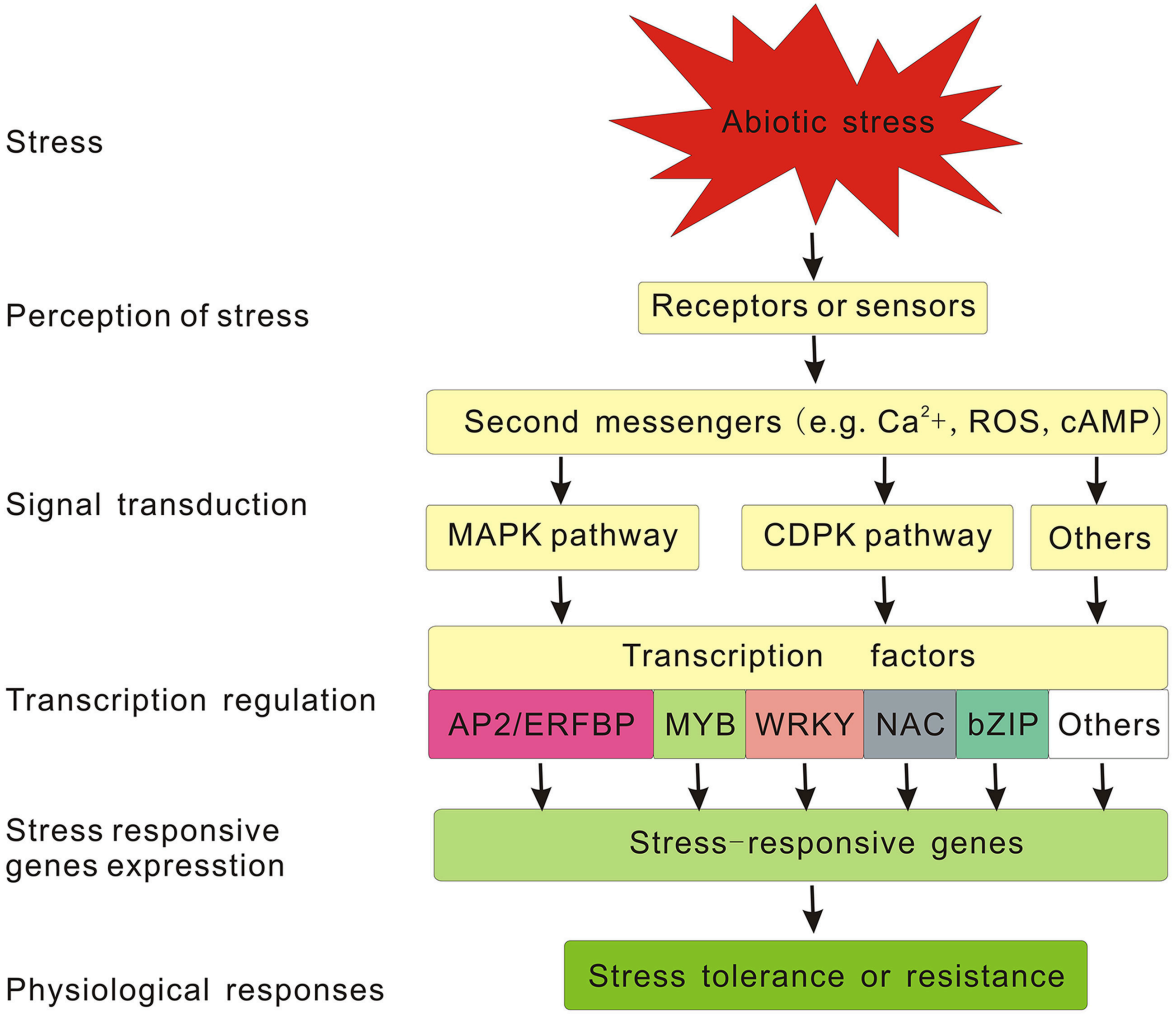
**Generic signaling pathway involved in plant abiotic stress responses**.

As sessile organisms, plants have evolved various defense mechanisms at multiple levels to respond to unfavorable environment including diverse abiotic stresses. As stated before, it is imperative to dissect regulatory mechanisms of stress response and identify the key regulators involved in this process to breed or genetically engineer stress-tolerant plants. With the availability of plant genomes and various omics tools including genomics, transcriptomics, and proteomics tools, major progress has been made in deciphering the stress signaling pathways and relevant components involved in plant abiotic stress response, but there is still much more to be determined (Liu J.-H. et al., 2014). According to our current knowledge about stress signaling pathways, the generic signaling pathway for any given abiotic stress can be divided into the following major steps: signal perception, signal transduction, stress responsive gene expression, in turn the activation of physiological, and metabolic responses (Chaves et al., 2003; Yamaguchi-Shinozaki and Shinozaki, 2006; Pérez-Clemente et al., 2013). In this process, plant cells first perceive stress stimulus through sensors or receptors located in the cell wall or membrane. Then the captured extracellular signals are converted into intracellular ones through second messengers including calcium ions, inositol phosphate, reactive oxygen species (ROS), cyclic nucleotides (cAMP and cGMP), sugars, and nitric oxide. Subsequently, these second messengers initiate the corresponding signaling pathways to transduce the signals (Chaves et al., 2009; Bhargava and Sawant, 2013). In many signal transduction pathways, the phosphorylation, and dephosphorylation of proteins mediated by protein kinase and phosphatases, respectively, is an important and effective mechanism for signal relay (Singh et al., 2003). For example, the mitogen activated protein kinases (MAPKs) pathway and calcium-dependent protein kinases (CDPKs) pathway are known to be involved in plant abiotic stress responses (Schaller et al., 2008; Huang G.T. et al., 2012). At the end of the phosphorylation cascade, TFs are activated or suppressed by protein kinases or phosphatases, and they further bind specifically to cis-elements in the promoters of stress-responsive genes and regulate their transcription (Danquah et al., 2014). Meanwhile, TFs themselves are regulated at the transcription level by other upstream components (Hirayama and Shinozaki, 2010) and also subjected to various tiers of modifications at the post-transcription level, such as ubiquitination and sumoylation, thus forming a complex regulatory network to modulate the expression of stress responsive genes, which in turn determine the activation of physiological and metabolic responses (Dong et al., 2006; Miura et al., 2007; Mizoi et al., 2013). All the components mentioned above, from the foremost receptors to the downstream functional genes, constitute the generic pathway for plant abiotic stress signal transduction (Figure 1). As one of the most important regulators, TFs function as terminal transducers and directly regulate the expression of an array of downstream genes by interacting with the specific cis-elements in their promoter region (Yamaguchi-Shinozaki and Shinozaki, 2006). In the last few decades, considerable research has been conducted to identify and characterize various TFs involved in plant abiotic stress responses either in abscisic acid (ABA)-dependent pathway or ABA-independent pathway, such as *AP2/EREBP, MYB, WRKY, NAC, bZIP*, and so on (Vinocur and Altman, 2005; Umezawa et al., 2006; Golldack et al., 2011). Numerous efforts have been also made to improve plant stress tolerance by engineering these TF genes, and some promising results have been reported in succession (Table 1). In the following sections, we mainly summarize current information on several major TF families including their features, roles, and biotechnological uses for improving the abiotic stress tolerance in plants.

**Table 1:** **Some examples of transgenic plants over-expressing transcription factor genes in recent years**.

**Family**	**Gene**	**Donor**	**Acceptor**	**Enhanced tolerance**	**References**
*AP2/ERFBP*	*LcDREB3a*	*Leymus chinensis*	*Arabidopsis*	Drought and salinity↑	Peng et al., 2013
	*LcDREB2*	*Leymus chinensis*	*Arabidopsis*	Salinity↑	Peng et al., 2013
	*LcERF054*	*Lotus corniculatus*	*Arabidopsis*	Salinity↑	Sun et al., 2014
	*VrDREB2A*	*Vigna radiata*	*Arabidopsis*	Drought and salinity↑	Chen et al., 2015
	*GmERF3*	*Glycine max*	*Tobacco*	Drought and salinity↑	Zhang et al., 2009
	*GmERF7*	*Glycine max*	Tobacco	Salinity↑	Zhai et al., 2013
	*SsDREB*	*Suaeda salsa*	Tobacco	Drought and salinity↑	Zhang X. et al., 2015
	*JERF3*	*Solanum lycopersicum*	Rice	Drought↑	Zhang et al., 2010
	*OsDREB2A*	*Oryza sativa*	Rice	Drought and salinity↑	Mallikarjuna et al., 2011
	*OsERF4a*	*Oryza sativa*	Rice	Drought↑	Joo et al., 2013
	*AtDREB1A*	*Arabidopsis*	Rice	Drought↑	Ravikumar et al., 2014
	*TaERF3*	*Triticum aestivum*	Wheat	Drought and salinity↑	Rong et al., 2014
	*TaPIE1*	*Triticum aestivum*	Wheat	Cold↑	Zhu et al., 2014
	*EaDREB2*	*Erianthus arundinaceus*	Sugarcane	Drought and salinity↑	Augustine et al., 2015
	*StDREB1*	*Solanum tuberosum*	Potato	Salinity↑	Bouaziz et al., 2013
*MYB*	*AtMYB15*	*Arabidopsis*	*Arabidopsis*	Drought and salinity↑	Ding et al., 2009
	*LcMYB1*	*Leymus chinensis*	*Arabidopsis*	Salinity↑	Cheng et al., 2013b
	*GmMYBJ1*	*Glycine max*	*Arabidopsis*	Drought and cold↑	Su et al., 2014
	*TaMYB3R1*	*Triticum aestivum*	*Arabidopsis*	Drought and salinity↑	Cai et al., 2015
	*TaPIMP1*	*Triticum aestivum*	Tobacco	Drought and salinity↑	Liu et al., 2011b
	*SbMYB2*	*Scutellaria baicalensis*	Tobacco	NaCl, mannitol, and ABA stresses↑	Qi et al., 2015
	*SbMYB7*	*Scutellaria baicalensis*	Tobacco	NaCl, mannitol, and ABA stresses↑	Qi et al., 2015
	*LeAN2*	*Lycopersicum esculentum*	Tobacco	Chilling and oxidative stresses↑	Meng et al., 2014
	*LeAN2*	*Lycopersicum esculentum*	Tomato	Heat↑	Meng et al., 2015
	*AtMYB44*	*Arabidopsis*	Soybean	Drought and salinity↑	Seo et al., 2012
	*OsMYB2*	*Oryza sativa*	Rice	Drought, cold, and salinity↑	Yang et al., 2012
	*OsMYB91*	*Oryza sativa*	Rice	Salinity↑	Zhu et al., 2015
	*MdSIMYB1*	*Malus × domestica*	Apple	Drought, cold, and salinity↑	Wang et al., 2014
*WRKY*	*AtWRKY28*	*Arabidopsis*	*Arabidopsis*	Salinity↑	Babitha et al., 2013
	*OsWRKY45*	*Oryza sativa*	*Arabidopsis*	Drought and salinity↑	Qiu and Yu, 2009
	*TaWRKY79*	*Triticum aestivum*	*Arabidopsis*	Drought↑	Qin et al., 2013
	*VvWRKY11*	*Vitis vinifera*	*Arabidopsis*	Drought↑	Liu et al., 2011a
	*ZmWRKY33*	*Zea may*	*Arabidopsis*	Salinity↑	Li et al., 2013
	*GhWRKY34*	*Gossypium hirsutum*	*Arabidopsis*	Salinity↑	Zhou et al., 2015
	*GsWRKY20*	*Glycine soja*	*Arabidopsis*	Drought↑	Luo et al., 2013
	*TaWRKY79*	*Triticum aestivum*	*Arabidopsis*	Salinity and ionic stress↑	Qin et al., 2013
	*TaWRKY93*	*Triticum aestivum*	*Arabidopsis*	Salinity, drought, and low temperature↑	Qin et al., 2015
	*TaWRKY10*	*Triticum aestivum*	Tobacco	Drought and salinity↑	Wang et al., 2013
	*GhWRKY39*	*Gossypium hirsutum*	Tobacco	Salinity↑	Shi et al., 2014
	*BdWRKY36*	*Brachypodium distachyon*	Tobacco	Drought↑	Sun et al., 2015
	*ZmWRKY58*	*Zea may*	Rice	Drought and salinity↑	Cai et al., 2014
	*MtWRKY76*	*Medicago truncatula*	*Medicago truncatula*	Drought and salinity↑	Liu et al., 2016
*NAC*	*ANAC019*	*Arabidopsis*	*Arabidopsis*	Cold↑	Jensen et al., 2010
	*ONAC063*	*Oryza sativa*	*Arabidopsis*	Salinity and osmotic tolerance↑	Yokotani et al., 2009
	*GmNAC20*	*Glycine max*	*Arabidopsis*	Salinity and freezing tolerance↑	Hao et al., 2011
	*ZmSNAC1*	*Zea may*	*Arabidopsis*	Cold, salinity, and drought↑	Lu et al., 2012
	*BnNAC5*	*Brassica napus*	*Arabidopsis*	Salinity↑	Zhong et al., 2012
	*TaNAC67*	*Triticum aestivum*	*Arabidopsis*	Cold, salinity, and drought↑	Mao et al., 2014
	*TaNAC29*	*Triticum aestivum*	*Arabidopsis*	Drought and salinity↑	Huang et al., 2015
	*MLNAC5*	*Miscanthus lutarioriparius*	*Arabidopsis*	Drought and cold↑	Yang et al., 2015
	*TaNAC2a*	*Triticum aestivum*	Tobacco	Drought	Tang et al., 2012
	*AhNAC3*	*Arachis hypogaea*	Tobacco	Drought↑	Liu et al., 2013
	*SNAC1*	*Oryza sativa*	Wheat	Drought and salinity↑	Saad et al., 2013
	*OsNAP*	*Oryza sativa*	Rice	Cold, salinity, and drought↑	Chen et al., 2014
*bZIP*	*ABP9*	*Zea may*	*Arabidopsis*	Drought, salinity, and cold↑	Zhang et al., 2011
	*GmbZIP1*	*Glycine max*	*Arabidopsis*	Drought, salinity, and cold↑	Gao et al., 2011
	*ZmbZIP72*	*Zea may*	*Arabidopsis*	Drought and salinity↑	Ying et al., 2012
	*TabZIP60*	*Triticum aestivum*	*Arabidopsis*	Drought, salt, and freezing tolerance↑	Zhang L. et al., 2015
	*PtrABF*	*Poncirus trifoliata*	Tobacco	Drought↑	Huang et al., 2010
	*GmbZIP1*	*Glycine max*	Tobacco	Drought, salinity, and cold↑	Gao et al., 2011
	*LrbZIP*	*Nelumbo nucifera*	Tobacco	Salinity↑	Cheng et al., 2013a
	*OsbZIP71*	*Oryza sativa*	Rice	Drought and salinity↑	Liu C. et al., 2014

## AP2/EREBP transcription factors

AP2/ERFBP family includes a large group of plant-specific TFs and is characterized by the presence of the highly conserved *AP2*/ethylene-responsive element-binding factor (ERF) DNA-binding domain that directly interact with GCC box and/or dehydration-responsive element (DRE)/C-repeat element (CRT) cis-acting elements at the promoter of downstream target genes (Riechmann and Meyerowitz, 1998). AP2/ERFBP TFs perform a variety of roles in plant developmental processes and stress responses, such as vegetative and reproductive development, cell proliferation, abiotic and biotic stress responses, and plant hormone responses (Nakano et al., 2006; Licausi et al., 2010; Sharoni et al., 2011). Presently, a multitude of AP2/ERFBP members have been identified in various species by means of genome-wide analysis, such as 145 in *Arabidopsis* (Riechmann and Meyerowitz, 1998), 163 in rice (Sharoni et al., 2011), 200 in poplar (Zhuang et al., 2008), 291 in *Chinese cabbage* (Song et al., 2013), 171 in *foxtail millet* (Lata et al., 2014), 116 in moso bamboo (Wu et al., 2015). Based on the number and similarity of *AP2/ERF* domains, these *AP2/EREBP* TFs are grouped into four major subfamilies: *AP2* (Apetala 2), *RAV* (related to *ABI3/VP1*), *DREB* (dehydration-responsive element-binding protein), and *ERF* (Sakuma et al., 2002; Sharoni et al., 2011). Among these, both *ERF* and *DREB* subfamilies have been extensively studied due to their involvement in plant responses to biotic and abiotic stresses.

The DREB subfamily can regulate the expression of multiple dehydration/cold-regulated (*RD/COR*) genes by interacting with *DRE/CRT* cis-elements (A/GCCGAC) located in the promoters of *RD/COR* genes that are responsive to water deficit and low-temperature, such as *COR15A, RD29A/COR78*, and *COR6.6* (Stockinger et al., 1997; Liu et al., 1998; Lucas et al., 2011). A lot of DREB-type TFs have been tested in many plants including *Arabidopsis*, wheat, tomato, soybean, rice, maize, and barley (Agarwal et al., 2006; Lata and Prasad, 2011; Mizoi et al., 2012). According to the variation in some conserved motifs and biological functions in divergent species, *DREB* TFs are further classified into two major subgroups: *DREB1/C*-repeat-binding factor (*DREB1/CBF*) and *DREB2*, and each of them is involved in separate signal transduction pathway under abiotic stresses (Dubouzet et al., 2003). Commonly, *DREB1/CBF* genes are involved in low temperature stress responses in *Arabidopsis* and rice, while *DREB2* genes respond to dehydration, high salinity and heat shock (Liu et al., 1998; Sakuma et al., 2002; Lucas et al., 2011). For example, three major *DREB1/CBF* members in *Arabidopsis, DREB1A/CBF3, DREB1B/CBF1* and *DREB1C/CBF2* are rapidly induced in response to cold stress (Stockinger et al., 1997; Gilmour et al., 1998; Liu et al., 1998; Shinwari et al., 1998). Over-expressing any one of these three *DREB1s/CBFs* displayed significantly improved tolerance to freezing, drought, and high salinity in transgenic *Arabidopsis* (Gilmour et al., 1998; Jaglo-Ottosen et al., 1998; Liu et al., 1998). Further, over-expressing *Arabidopsis DREB1/CBF* genes improved freezing tolerance in oilseed rape (Jaglo et al., 2001) and chilling tolerance in tomato, tobacco and rice (Tsai-Hung et al., 2002; Kasuga et al., 2004; Ito et al., 2006). Some *DREB1/CBF* homologous genes have also been isolated from many other plant species including tomato, oilseed rape, wheat, rye, rice, and maize, and some of them have been used to produce transgenic plants with improved tolerance to abiotic stress (Jaglo et al., 2001; Dubouzet et al., 2003; Qin et al., 2004). In contrast, *DREB2* genes have been studied in a limited number of plant species, but the existing studies have shown that *DREB2* genes are also involved in abiotic stress responses in plants. In *Arabidopsis, DREB2A* and *DREB2B* are major *DREB2s* induced by dehydration, high salinity, and heat, while *DREB2C* is induced by heat later than them (Liu et al., 1998; Nakashima et al., 2000; Sakuma et al., 2006b; Lim et al., 2007). Over-expression of the constitutively active form of *AtDREB2A* from *Arabidopsis* improved the tolerance to drought and osmotic stress in transgenic *Arabidopsis* plants (Sakuma et al., 2006a; Xu et al., 2008b). Over-expression of *ZmDREB2A* from maize enhanced drought tolerance in transgenic *Arabidopsis* plants (Qin et al., 2007). The transgenic plants harboring *GmDREB2* from soybean also showed enhanced tolerance to drought and high salinity without any growth retardation (Chen et al., 2007), as was observed in transgenic rice by over-expressing *OsDREB2A* under control of stress-inducible *RD29A* promoter (Mallikarjuna et al., 2011).

The ERF subfamily is the largest group of the AP2/EREBP TF family (Dietz et al., 2010) and functions in plant stress tolerance by regulating the stress-responsive genes through interacting with the cis-element GCC boxes with core sequence of AGCCGCC (Ohme-Takagi and Shinshi, 1995; Hao et al., 1998). An array of ERF genes are induced by various abiotic stresses, such as drought, high salinity, osmotic stress, and cold (Xu et al., 2008a). Over-expression of these ERF genes resulted in improvement of abiotic stress tolerance in transgenic plants, as summarized in Table 1. It is worth mentioning that some ERFs function in both biotic and abiotic stress tolerance, and this is partly due to their involvement in various hormonal signaling pathways including ethylene, JA, or SA (Liang et al., 2008). For example, over-expressing *TaPIE1* in wheat significantly enhanced resistance to both pathogen and freezing stress (Zhu et al., 2014). Over-expressing *GmERF3* in tobacco not only enhanced resistance against infection by pathogen and tobacco mosaic virus (TMV) but also improved tolerance to high salinity and dehydration (Zhang et al., 2009). So far, functions of a limited number of *ERFs* have been well characterized, but most of *ERF* family members have yet to be identified.

## MYB transcription factors

The MYB TFs are widely distributed in plants and form a large family characterized by a highly conserved MYB domain for DNA-binding, which contains from 1 to 4 imperfect repeats (*MYB* repeat) at the N-terminus. In contrast, the activation domain is located at the C-terminus and varies significantly among MYBs, leading to versatile regulatory roles of *MYB* family. According to the number of MYB domain repeats, the MYB TFs are divided into four groups: 1R-MYB (MYB-related type), R2R3-MYB, R1R2R3-MYB, and 4R-MYB, containing one, two, three, and four *MYB* repeats, respectively. Among them, the R2R3-MYBs are more prevalent in plants (Dubos et al., 2010; Ambawat et al., 2013; Li et al., 2015). So far, large numbers of MYB members have been identified in different plant species, such as 198 in *Arabidopsis* (Yanhui et al., 2006), 183 in rice (Yanhui et al., 2006), 229 in apple (Cao et al., 2013), 177 in sweet orange (Hou et al., 2014), 209 in *foxtail millet* (Muthamilarasan et al., 2014).

Numerous MYB TFs have been found to function in many significant physiological and biochemical processes including cell development and cell cycle, primary and secondary metabolism, hormone synthesis and signal transduction, as well as in plant responses to various biotic and abiotic stresses (Allan et al., 2008; Dubos et al., 2010; Ambawat et al., 2013). Recently, some abiotic stress-responsive *MYB* TFs in *Arabidopsis* and other plants have been well summarized by Li (Li et al., 2015). For example, *AtMYB15* improved freezing tolerance by regulating *CBF* expression (Agarwal et al., 2006); *AtMYB44, AtMYB60*, and *AtMYB61* improved drought tolerance by regulating stomatal movement (Cominelli et al., 2005; Liang et al., 2005; Jung et al., 2008). Especially, *AtMYB96* improved drought tolerance either by integrating ABA and auxin signals (Seo et al., 2009) or by activating cuticular wax biosynthesis (Seo et al., 2011), and also improved freezing and drought tolerance by regulating a lipid-transfer protein LTP3. This fact shows that a MYB factor can regulate diverse target genes involved in various physiological processes under abiotic stresses. In addition, *OsMYB2* from rice was induced by salt, cold, and dehydration stress. The transgenic plants with over-expressing *OsMYB2* exhibited enhanced tolerance to various stresses by the alteration of expression levels of numerous genes involving diverse functions in stress response (Yang et al., 2012). Salt and freezing tolerance in *Arabidopsis* was significantly enhanced by over-expressing either *GmMYB76* or *GmMYB177* from soybean (Liao et al., 2008).

## WRKY transcription factors

The WRKY family is also extensively distributed in plants and contains many members. WRKY TFs are characterized by the presence of one or two highly conserved WRKY domains of about 60 amino acid residues, which contains a conserved WRKYGQK motif at the N-terminus and a C2H2 or C2HC zinc-finger motif at the C-terminus The WRKY domains can specifically bind to W-box cis-elements with a core sequence of TTGACC/T, located at the promoters of many target genes. According to the number of WRKY domains and the feature of the zinc finger motif, the WRKY TFs can be categorized into three groups. Group I members contain two WRKY domains and a C2H2 zinc-finger motif, whereas group II and III members contain one WRKY domain with a C2H2 and C2HC zinc-finger motif, respectively (Eulgem et al., 2000; Ulker and Somssich, 2004; Pandey and Somssich, 2009; Rushton et al., 2010). Since the cloning of the first cDNA encoding a WRKY protein (SPF1) from sweet potato (Ishiguro and Nakamura, 1994), an increasing number of WRKY TFs have been identified in various plants, such as 74 in *Arabidopsis* (Ulker and Somssich, 2004), 102 in rice (Wu et al., 2005), 104 in poplar (He et al., 2012), 86 in *Brachypodium distachyon* (Wen et al., 2014), 182 in soybean (Bencke-Malato et al., 2014), and 116 and 102 genes in two different species of cotton (Dou et al., 2014).

WRKY TFs have been shown to participate in various processes in plants, including plant growth, seed development, leaf senescence, and responses to biotic and abiotic stresses (Rushton et al., 2010). Accumulating evidence has demonstrated that WRKY TFs play key roles in plant responses to a variety of abiotic stresses such as drought, salt, heat, cold, and osmotic pressure, and these topics have been extensively reviewed recently (Chen et al., 2012; Rushton et al., 2012; Tripathi et al., 2014; Banerjee and Roychoudhury, 2015). Over-expression of some stress-responsive *WRKY* genes showed enhanced tolerance to abiotic stresses in transgenic plants. For example, transgenic rice plants harboring *OsWRKY11* gene showed significant heat and drought tolerance (Wu et al., 2009). Transgenic *Arabidopsis* plants over-expressing *GmWRKY21* gene exhibited improved tolerance to cold stress, while over-expressing *GmWRKY54* gene improved tolerance to drought and salt stress (Zhou et al., 2008). Transgenic *Arabidopsis* plants over-expressing *VvWRKY11* improved to tolerance mannitol-induced osmotic stress (Liu et al., 2011a). Although some WRKYs in several plants have been functionally characterized, the vast majority of WRKYs in many plants, especially in non-model plants, are far from being functionally elucidated.

## NAC transcription factors

Like the transcription factor families mentioned above, the NAC TFs also comprise a large plant-specific superfamily present in a wide range of plant species. The typical features of a NAC TF contain a highly conserved NAC domain in the N-terminal region and a variable transcriptional regulatory region in the C-terminal region. The NAC domain is associated with DNA binding, nucleus-oriented localization, and the formation of homodimers or heterodimers with other NAC proteins, while the transcriptional regulatory functions as a transcriptional activator or repressor (Olsen et al., 2005; Puranik et al., 2012). NAC TFs can regulate the transcription of downstream target genes by interacting with *NAC* recognition sequence (NACRS) with the CACG core-DNA binding motif in the promoter of these genes. NAC TFs have been found to participate in various processes including flower development, formation of secondary walls and cell division, shoot apical meristem formation, leaf senescence, as well as biotic and abiotic stress responses (Olsen et al., 2005; Tran et al., 2010; Nakashima et al., 2012; Nuruzzaman et al., 2013; Banerjee and Roychoudhury, 2015). To date, a lot of putative NAC TFs have been identified in many sequenced species at genome-wide scale, such as 117 in *Arabidopsis* and 151 in rice (Nuruzzaman et al., 2010), 152 in soybean (Le et al., 2011), 204 in Chinese cabbage (Liu T.K. et al., 2014), 152 in maize (Shiriga et al., 2014), tomato (Su et al., 2015). Moreover, quite a lot of them have been found to be involved in abiotic stress responses. For instance, 33 *NAC* genes changed significantly in *Arabdopsis* under salt stress (Jiang and Deyholos, 2006), 40 *NAC* genes changed under drought or salt stress in rice (Fang et al., 2008), 38 *NAC* genes changed in soybean under drought stress (Le et al., 2011), 32 *NAC* genes responded to at least two kinds of treatments in *Chrysanthemum lavandulifolium* (Huang H. et al., 2012). These stress-responsive *NAC* genes showed differential expression patterns such as tissue-specific, developmental stage- or stress-specific expression, indicating their involvement in the complex signaling networks during plant stress responses. Some of these stress-responsive *NAC* genes have been over-expressed in *Arabidopsis*, rice and other plants and displayed positive effects, summarized in Table 1.

## bZIP transcription factors

The basic leucine zipper (bZIP) family contains a conserved bZIP domain which is composed of a highly basic region for nuclear localization and DNA binding at the N-terminus and a leucine-rich motif for dimerization at the C-terminus (Landschulz et al., 1988; Hurst, 1994). Like other TFs, the bZIP TFs not only play pivotal roles in developmental processes but also respond to various abiotic stresses such as drought, high salinity, and cold stresses (Jakoby et al., 2002). Now, many members of the bZIP TF family have been identified or predicted at genome-wide level in some species. For example, it has been reported 75 in *Arabidopsis* (Jakoby et al., 2002), 89 in rice (Nijhawan et al., 2008), 125 in maize (Wei et al., 2012), 89 in barley (Pourabed et al., 2015), 55 in grapevine (Liu J. et al., 2014), 96 in *Brachypodium distachyon* (Liu and Chu, 2015). However, only a small portion of bZIP TFs has been well studied and most studies on their involvement in stress responses have shown that bZIP TFs are induced by ABA and regulate the expression of stress-related genes in ABA-dependent manner through interaction with specific ABA-responsive cis-acting elements (ABRE) in their promoter region (Uno et al., 2000; Kim et al., 2006; Zou et al., 2008). A lot of efforts have been made to improve abiotic stress tolerance in transgenic plants by over-expressing some stress-responsive bZIP genes and some successful example have been achieved, as listed in Table 1.

## Conclusions and perspectives

Taking five large families of TFs as examples, this review emphasizes the promising roles of TFs as tools to improve plant responses to multiple abiotic stresses. In addition to the above-mentioned several TF families, there are still other TF families such as DNA binding with one finger (Dof) TFs, basic helix-loop-helix (bHLH) TFs, homeodomain-leucine zipper (HD-Zip) TFs, heat shock TFs (HSFs), etc. How to select the key TFs in such a huge gene families and fully display its potential is still an important issue before us. Although a great deal of information about TFs has been accumulated on their involvement in response to diverse abiotic stresses and a good number of promising candidate TF genes have been validated, but there are still some problems to be solved. First, functional redundancy between different TF members may hinder the dissection of the functions of an individual member. Second, most of transgenic studies based on TFs focused on plant growth and tolerance to a given stress at a given developmental stage rather than whole stage. Moreover, the evaluation of transgenic plants was conducted in controlled laboratory or greenhouse conditions rather than field conditions. Third, the constitutive over-expression of some TF genes may improve the stress tolerance, but occasionally lead to negative effects in transgenic plants such as dwarfing, late flowering, and lower yields. Finally, the complete regulation mechanism of individual transcription factor including its upstream and downstream co-regulators, as well as their interactions remains largely unknown.

Abiotic stress response in plants is an extremely complicated process because of the huge gene families and the complex interactions between TFs and cis-elements on the promoters of target genes. Moreover, one transcription factor may regulate a vast array of target genes with the corresponding cis-elements on the promoters, whereas one gene with several types of cis-elements may be regulated by different families of TFs. Thus, the stress-responsive TFs not only function independently but also cross-talk between each other in response to various abiotic stress responses, which indicates the complexity of signaling networks involved in plant stress responses. In the future research, we should first identify multiple stress-responsive TF genes by comparing their expression patterns and the identification of commonly regulated genes which have been proposed to be required for universal stress responses or represent points of cross-talk between signaling pathways (Prasch and Sonnewald, 2015). Genetic manipulation of these identified genes should be a more powerful approach for improving plant tolerance to multiple stresses than manipulation of individual functional genes. Then, the selected TF genes should be validated not only in model plant species but also in crop plants by use of stress-inducible promoter which can minimize the negative effects caused by over-expressing some TF genes. Furthermore, the critical field trials are required to evaluate the transgenetic plants, especially focusing on their growth and tolerance in the whole life period. which is a necessary step in many strategies to develop stress-tolerant crops. Taken together, we still need to struggle for a complete understanding the precise regulatory mechanisms involved in plant abiotic stress responses, which helps to obtain the promising candidate TF genes for breeding multiple abiotic stress-tolerant crops with better yields and qualities.

## Author contributions

HYW, HLW, XT wrote the paper. HS provided the paper frame and revised the final paper. All authors reviewed the final manuscript.

### Conflict of interest statement

The authors declare that the research was conducted in the absence of any commercial or financial relationships that could be construed as a potential conflict of interest.
